# *Lactobacillus rhamnosus* LB1 Alleviates Enterotoxigenic *Escherichia coli*-Induced Adverse Effects in Piglets by Improving Host Immune Response and Anti-Oxidation Stress and Restoring Intestinal Integrity

**DOI:** 10.3389/fcimb.2021.724401

**Published:** 2021-11-02

**Authors:** Tao Wu, Yutao Shi, Yanyan Zhang, Min Zhang, Lijuan Zhang, Zhipeng Ma, Di Zhao, Lei Wang, Hai Yu, Yongqing Hou, Joshua Gong

**Affiliations:** ^1^ Hubei Key Laboratory of Animal Nutrition and Feed Science, School of Animal Science and Nutrition Engineering, Wuhan Polytechnic University, Wuhan, China; ^2^ Guelph Research and Development Centre, Agriculture and Agri-Food Canada, Guelph, ON, Canada

**Keywords:** *Lactobacillus rhamnosus*, ETEC, gut health, gut functions, piglets

## Abstract

Enterotoxigenic *Escherichia coli* (ETEC) is a common enteric pathogen that causes diarrhoea in humans and animals. *Lactobacillus rhamnosus* LB1 (formerly named *Lactobacillus zeae* LB1) has been shown to reduce ETEC infection to *Caenorhabditis elegans* and *Salmonella* burden in pigs. This study was to evaluate the effect of *L. rhamnosus* LB1 on the gut health of lactating piglets that were challenged with ETEC. Six-four piglets at 7 days of age were equally assigned into 8 groups (8 piglets per group): 1) control group (basal diet, phosphate buffer saline); 2) CT group (basal diet + 40 mg/kg colistin); 3) LL group (basal diet + 1 × 10^7^ CFU/pig/day LB1); 4) HL group (basal diet + 1 × 10^8^ CFU/pig/day LB1); 5) ETEC group: (basal diet + ETEC challenged); 6) CT + ETEC group (basal diet + CT + ETEC); 7) LL + ETEC group (basal diet + 1 × 10^7^ CFU/pig/day LB1 + ETEC); 8) HL + ETEC group (basal diet + 1 × 10^8^ CFU/pig/day LB1 + ETEC). The trial lasted ten days including 3 days of adaptation. Several significant interactions were found on blood parameters, intestinal morphology, gene, and protein expression. ETEC infection disrupted the cell structure and biochemical indicators of blood, undermined the integrity of the intestinal tract, and induced oxidative stress, diarrhoea, intestinal damage, and death of piglets. The supplementation of *L. rhamnosus* LB1 alleviated ETEC’s adverse effects by reducing pig diarrhoea, oxidative stress, and death, modulating cell structure and biochemical indicators of blood, improving the capacity of immunity and anti-oxidation stress of pigs, and restoring their intestinal integrity. At the molecular level, the beneficial effects of *L. rhamnosus* LB1 appeared to be mediated by regulating functional related proteins (including HSP70, Caspase-3, NLRP3, AQP3, and AQP4) and genes (including RPL4, IL-8, HP, HSP70, Mx1, Mx2, S100A12, Nrf2, GPX2 and ARG1). These results suggest that dietary supplementation of *L. rhamnosus* LB1 improved the intestinal functions and health of piglets.

## Introduction

Probiotics including lactobacilli have long been used to improve animal/human gut health that are relevant to the host health, nutrition, and well-being. Antagonizing enteric infections and enhancing host physiological and immune responses are among the crucial benefits of probiotics, in which the modulation of the gut micbrobiota and its metabolites is one of the mechanisms (Matsuzaki, 2000; Lebeer et al., 2010). Enterotoxigenic *Escherichia coli* (ETEC) is a common enteric pathogen causing diarrhoea. It is one of the most important diseases in young farm animals and also the second leading cause of death in children under five years old (Bin et al., 2018; Zhang et al., 2021). The ETEC induced diarrhoea in piglets mainly one week after weaning, resulting in considerable economic losses to the swine industry due to increased morbidity and mortality as well as reduced growth performance (Kazazian and Moran, 2017; Rodic, 2017). ETEC has been controlled by the prophylactic use of antibiotics in the past (Zhou et al., 2014). However, there has been an increasing concern over the widespread of antibiotic resistance potentially lined to the use of in-feed antibiotics, which poses a threat to public health and food safety. It thus becomes imperative to develop effective alternatives to in-feed antibiotics (Economou and Gousia, 2015).

Our previous studies have shown that a *Lactobacillus* isolate (*L. zeae* LB1) was effective in reducing either ETEC or *Salmonella* infection in *Caenorhabditis elegans* (Wang C. et al., 2011; Zhou et al., 2014). In addition, it could lessen *Salmonella* infection in both chickens and post-weaning pigs (Yang et al., 2014; Yin et al., 2014). We hypothesized that *L. zeae* LB1 could also protect piglets from ETEC infection. Therefore, this research was to study the beneficial effect of *L. zeae* LB1 supplementation on the gut health of piglets in lactation that had been challenged with ETEC *via* the analyses of intestinal morphology, blood parameters and redox status at both animal and molecular levels. Recently, the isolate was reclassified as *Lactobacillus rhamnosus*, based on the examination of bacterial morphology, cultural growth, substrate utilization/fermentation properties and DNA sequence homology of the entire 16S rRNA gene. All the results are reported herein.

## Materials and Methods

### Bacteria and Cultures

*L. rhamnosus* LB1 was from the Guelph Research and Development Centre, Agriculture and Agri-Food Canada. This isolate was formerly named *L. zeae* LB1 based on the partial sequences of 16S rRNA gene and BLAST analysis then (Wang C. et al., 2011). Recently, the isolate was identified as *L. rhamnosus* by the Guangdong Microbial Culture Centre, China (TESTING CNAS L1747; Report No.: 2020JZ0010R01D) through systematic microbiological analyses. The isolate shows 100% sequence homology of 16S rDNA with *L. rhamnosus*. Gram-positive, rod shape and no spore production. On the MRS (de Man, Rogosa and Sharpe) agar plate, its colonies appear to be milky white, smooth surface, opaque, convex and edge neat. The isolate also demonstrates growth and substrate utilization/fermentation properties of *L. rhamnosus* (**Table S1**). To prepare the inoculum of *L. rhamnosus* LB1 for the animal trial, the bacterium was anaerobically cultured in MRS broth at 37°C. After 16 hours of incubation, LB1 cells were collected by centrifugation at 6,000 g for 5 min and then resuspension in PBS (1 × 10^8^ CFU/ml; OD_600_ = 0.5). The ETEC isolate, K88ac (C83715), was from China Institute of Veterinary Drug Control, Beijing, China, which has been used as a standard strain (Xu et al., 2020). The pathogenicity of ETEC depends on its colonization through F4 fimbriae (*faeG*) and production of STa, STb, and LT enterotoxins (Zhu et al., 2011). To prepare the inoculum of ETEC for the animal trial, the pathogen was cultured in Tryptic Soy Broth (TBS) at 37°C with agitation at 120 r/min. After 12-hour incubation, ETEC cells were collected by centrifugation at 6,000 g for 5 min and then resuspension in PBS (5 × 10^8^ CFU/ml; OD_600 =_ 0.8).

### Animals and Trial Design

The protocols of animal use and care conformed to the Guide for the Care and Use of Hubei Key Laboratory of Animal Nutrition and Feed Science and were approved by the Animal Care and Use Committee of Wuhan Polytechnic University (Wuhan, Hubei, China) (2018-0625, 25th Jun 2018). Sixty-four healthy crossbred piglets at 7 day of age (Duroc × Landrace × Yorkshire) were chosen and the commercially available milk replacer (D80 Poly milk, shanghai, China) was used as a basic diet. After 3-day adaptation, the piglets were assigned randomly, on the basis of body weight (BW) and litter origin, to 8 groups: (8 piglets/group): 1) control group - piglets fed the basal diet and treated with phosphate buffered saline (PBS, pH = 7.4); 2) CT group - piglets fed the basal diet supplemented with 40 mg/kg BW of colistin (CT; Zhongnong Pharmaceutical, Xinxiang, China); 3) LL group - piglets fed the basal diet supplemented with 1 × 10^7^ LB1/pig/day (relative low concentration of LB1, LL); 4) HL group - piglets fed the basal diet supplemented with 1×10^8^ CFU/pig/day LB1 (relative high concentration of LB1, HL); 5) ETEC group: piglets fed the basal diet, and challenged with 1 × 10^10^ CFU/pig ETEC; 6) CT + ETEC group - piglets fed the basal diet supplemented with 4 mg/kg BW of CT, and challenged with 1 × 10^10^ CFU/pig ETEC; 7) LL + ETEC group - piglets fed the basal diet supplemented with 1 × 10^7^ CFU/pig/day LB1, and challenged with 1 × 10^10^ CFU/pig ETEC; 8) HL + ETEC group - piglets fed the basal diet supplemented with 1 × 10^8^ CFU/pig/day LB1, and challenged with 1 × 10^10^ CFU/pig ETEC. Before the animal trial, faeces from each piglet was tested for ETEC by PCR analysis using primers specific to *faeG* (Zhu et al., 2011) and the method described by Choi et al. (2020). No ETEC was detected in the faecal samples.

The healthy crossbred piglets were housed individually in pens that were placed in a temperature controlled nursery barn (28-30°C). All diets were isocaloric. The trial lasted 10 days (D0-D9), including three days of adaptation (D0-D2). The treatment with PBS, colistin, or LB1 was from D3 to D7. ETEC challenge was carried out on D7. The trial ended on D9. From day 3, the colistin (40 mg/kg) and *L. rhamnosus* LB1 (1 × 10^7^ CFU and 1 × 10^8^ CFU) were separately mixed with 5 ml PBS and then orally fed to piglets at 15:00 each day during the trial. The doses of LB1 were selected by taking into account our previous administration of LB1 in chickens (Yang et al., 2014) and the animal body weight to be tested in this study. On day 7, ETEC was orally delivered to piglets at 5 × 10^9^ CFU/pig each time at 8:00 and 20:00, respectively, as described previously (Wu et al., 2018b). Animal growth performance including body weight and feed intake was recorded daily. Individual pigs were examined for diarrhoea on day 1 and day 2 of post-infection, four times per day. Diarrhoea was quantified by the following equation for each pig. Diarrhea rate (%) = total diarrhea incidences/8 × 100.

### Samples Collection

The D-xylose absorptive test was performed to measure intestinal absorption capacity and mucosal integrity prior to scarification of piglets, which was conducted on D9 of the trial with 50% piglets per groups. At 1 h after the oral administration of 10% D-xylose at the dose of 1 ml/kg BW, blood samples were collected from the anterior vena cava into heparinized vacuum tubes (Becton-Dickinson Vacutainer System, Franklin Lake, NJ, USA). Blood samples were centrifuged at 1300 g for 15 min at 4°C to obtain plasma, which was stored at −80°C until analysis.

After blood sampling, all pigs were killed under sodium pentobarbital anaesthesia (50 mg/kg BW, iv) in order to obtain the small intestine mucosal samples. The pig abdomen was opened immediately from sternum to pubis and the whole gastrointestinal tract was immediately exposed, the small intestine was immediately dissected free of the mesentery on a chilled stainless-steel tray, then the 5-cm segments were cut, respectively, at each distal duodenum, mid-jejunum and mid-ileum. The 5-cm segments were gently flushed with ice-cold PBS and placed in chilled formalin solution (10%), then processed by embedding and staining for the observation of intestinal morphology. All the samples were collected within 10 min. Intestinal tissues and digesta samples were stored at −80°C until analysis.

### Blood Parameters

Plasma biochemical indicators were measured with corresponding kits using a Hitachi 7060 Automatic Biochemical Analyzer (Hitachi, Tokyo, Japan), and blood cell counts were estimated using ADVIA^®^2120i Automatic Haematology System (Siemens Healthcare, Erlangen, Germany). The activities of diamine oxidase (DAO), total superoxide dismutase(T-SOD), glutathione peroxidase (GSH-PX), and catalase(CAT), and the content of D-xylose, malondialdehyde (MDA), and hydrogen peroxide (H_2_O_2_) were determined using commercially available kits (Jiancheng Bioengineering Institute, Nanjing, China).

### Histology of Intestinal Issues

Intestinal tissue samples for the morphometric study were processed as described previously (Wu et al., 2018a). Briefly, the issue samples were dehydrated and embedded in paraffin, sectioned at a thickness of 4 mm, and stained with haematoxylin and eosin. Morphological examination was conducted with a light microscope (Leica microsystems, Wetzlar, Germany) with the Leica Application Suite image analysis software (Leica microsystems, Wetzlar, Germany). Intestinal villus height and width, as well as crypt depth, were measured to calculate both the villus crypt ratio and villous surface area. For each intestinal region (duodenum, jejunum, ileum, and colon), 64 prepared tissue slides from each group of pigs were examined, in which eight scopes per slide were inspected in a double-blind manner.

### RNA Extraction and Reverse Transcription

Each frozen sample (approximately 100 mg) was powdered and homogenized, and total RNA was isolated using the Trizol Reagent protocol (Invitrogen, Carlsbad, CA). Total RNA was quantified using the NanoDrop ^®^ ND-1000A UV-VIS spectrophotometer (Thermo Scientific, Wilmington, DE, USA) at an OD of 260 nm, and its purity was assessed by determining the OD260/OD280 ratio (1.8 to 2.1). Total RNA was treated with gDNA Eraser (5 min at 42°C) to remove genomic DNA and removal of gDNA was confirmed by PCR using treated or untreated RNA samples as templates for a comparison. Reverse transcription was then carried out using a PrimeScript ^®^ RT reagent kit (Takara, Dalian, China) according to the manufacturer’s instruction. In brief, the total volume of the cDNA reaction was 20 μL. The reaction mixture contained 10.0 µL RNA, 1.0 µL PrimeScript RT Enzyme Mix 1, 4.0 µL RT Primer Mix, 4.0 µL 5 × PrimeScript Buffer 2, and 1.0 µL RNase Free dH_2_O. The cDNA reaction was performed under the following conditions (two-step amplification): 37°C for 15 min and then 85°C for 5 sec. Finally, the synthesized cDNA was stored at -20°C until use.

### Quantitative PCR Assay

The gene expression levels were quantitated using the method of quantitative PCR (qPCR) assays. The qPCR was carried out with primers (**Table 1**), which were either verified experimentally using the conditions published previously including the housekeeping gene encoding ribosomal protein L 4 (RPL4) or were designed using Primer Express software version 3.0 (Applied Biosystems). The qPCR was performed using the SYBR ^®^ Premix Ex Taq TM (Takara, Dalian, China) on an Applied Biosystems 7500 Fast Real-Time PCR System (Foster City, CA). The total volume of PCR reaction system was 50 μL, containing 0.2 µM of each primer, 25 µL of SYBR ^®^ Premix Ex Taq TM (2 ×), and 4 µL of cDNA. All PCR assays were performed in triplicate on a 96-well real-time PCR plate (Applied Biosystems) under the following conditions (two-step amplification): 95°C for 30 sec, followed by 40 cycles of 95°C for 5 sec and 55-65°C for 31 sec depending on the melting temperature of each pair of primers. A subsequent melting curve (95°C for 15 sec, 60°C for 1 min and 95°C for 15 sec) with continuous fluorescence measurement was constructed. Data were analysed using the 2^-ΔΔ^Ct method as described (Yi et al., 2016).

**Table 1:** Primers for qPCR analysis.

Genes	Forward	Reverse	Source of reference
RPL4	F:5’-GAGAAACCGTCGCCGAAT -3’	R: 5GCCCACCAGGAGCAAGTT’-3’	Yi et al., 2016
HP	F:5’- GTTCGCTATCACTGCCAAAC -3’	R:5’- CAGTTTCTCTCCAGTGACCT -3’	This study
HSP70	F:5’-GACGGAAGCACAGGAAGGA-3’	R:5’-GAAGACAGGGTGCGTTTGG-3’	Yi et al., 2016
S100A12	F:5’-TTGAAGGGTGAACGCAAGG-3’	R:5’-AATGCCCCAACCGAACTG-3’	This study
Mx1	F:5’-AGTGCGGCTGTTTACCAAG-3’	R:5’-TTCACAAACCCTGGCAACTC-3’	Yi et al., 2016
Mx2	F:5’-CGCATTCTTTCACTCGCATC-3’	R:5’-CCTCAACCCACCAACTCACA-3’	Yi et al., 2016
GPX2	F:5’-CAGGGCAGTGCTGATTGAGA-3’	R:5’-GCAAGGGAAGCCAAGAACC-3’	This study
ARG1	F:5’-GGCTGGTCTGCTTGAGAAAC-3’	R:5’-ATCGCCATACTGTGGTCTCC-3’	This study
Nrf2	F: 5’-GAAGTGATCCCCTGATGTTGC-3’	5’-ATGCCTTCTCTTTCCCCTATTTCT-3’	Wu et al., 2018a
IL-8	F: 5’-TTCGATGCCAGTGCATAAATA-3’	R: 5’-CTGTACAACCTTCTGCACCCA-3’	Zhao et al., 2017

### Western Blot Analysis

The protein expression levels were determined by Western blot analysis. The primary antibodies included: aquaporins 3 (AQP3, Santa Cruz Biotechnology, Shanghai, China), aquaporins 4 (AQP4, Santa Cruz Biotechnology, Shanghai, China), NLR family pyrin domain containing 3 (NLRP3, Invitrogen, Carlsbad, USA), Heat Shock Protein 70 (HSP70, Assay Designs, Inc., Michigan, USA), Caspase-3 (Cell Signalling Technology, Inc., Danvers, USA), and β-actin (Cell Signalling Technology, Inc., Danvers, USA). The secondary antibodies were: anti-rabbit (1: 10000) and anti-mouse (1:5000). The blots were visualized and analysed using a chemiluminescence kit (Amersham Biosciences, Uppsala, Sweden) and forming image system (Alpha Innotech, New York, USA).

### Statistical Analysis

The data were analysed by two way analysis of variance (ANOVA) using the MIXED procedure of SAS (SAS Release 9.4, SAS Institute Inc., Cary, NC, USA). ETEC, Probiotics, and interaction between ETEC and Probiotics (E x Pro) as fixed factors were included in a linear mixed model and pigs were treated as a random factor. Tukey–Kramer range test was processed as *post hoc* test to present the significant difference. A p-value of < 0.05 was considered statistically significant, and a p-value of < 0.10 was defined as a significant trend. When a p-value is < 0.001, the difference was regarded as extremely significant. All the assays were repeated at least twice.

## Results

### Growth Performance

During the experiment (D3-D7), the average daily feed intake (ADFI), average daily gain (ADG), diarrhoea rate (DR) and mortality rate (MR) were recorded (**Table 2**). The ETEC infection significantly reduced the ADG (*p* < 0.05). ADG and ADFI in the group of HL were significant increase (*p* < 0.05), while the LL group had a tendency to increase ADG and ADFI (*p* < 0.10). The supplementation of CT significantly improved ADG (*p* < 0.05) and exhibited a tendency to improve ADFI (*p* < 0.10). Compared with control group, DR in the ETEC group was significantly increased (*p* < 0.05). An extremely significant ETEC × probiotic interaction was found on the diarrhoea rate (*p* < 0.001). Compared with DR in the ETEC group, DR in the CT+ETEC, LL+ETEC and HL+ETEC groups had significantly reduced DR (*p* < 0.05). Additionally, a significant trend of ETEC × probiotics interaction was observed on ADFI (*p* < 0. 10).

**Table 2:** Effects of *Lactobacillus rhamnosus* LB1 on the growth performance of piglets..

Item	- ETEC				+ ETEC				SEM	ETEC		Pro				*P*-value		
	-	CT	LL	HL	-	CT	LL	HL		+	-	-	CT	LL	HL	E	Pro	E×Pro
ADG g	2.0	8.3	10.8	15.6	-36.9	16.7	-22.8	6.5	9.45	-9.08	9.18	-17.4^a^	12.5^b^	-6.0^ab^	11.1^b^	0.009	0.006	0.055
ADFI g	89.7	87.3	90.5	93.0	75.8	91.7	86.9	95.7	3.59	87.5	90.1	82.8^a^	89.5^ab^	88.7^ab^	94.3^b^	0.299	0.024	0.058
DR %	9.4^a^	17.2^a^	14.1^a^	12.5^a^	78.1^b^	31.3^a^	23.4^a^	20.3^a^	5.03	38.3	13.3	43.8	24.2	18.8	16.4	< 0.001	< 0.001	< 0.001
MR %	0.0	0.0	0.0	0.0	25.0	0.0	12.5	0.0	7.28	9.3	0.0	12.5	0.0	6.3	0.0	0.075	0.269	0.269

-: basal diet; CT: basal diet + antibiotic; LL: basal diet + low dose LB1; HL: basal diet + high dose LB1. SEM, standard error of mean (same in the following tables). Pro, probiotics (same in the following tables); ADG, average daily gain; ADFI, average daily feed intake; DR, diarrhoea rate; MR, mortality rate. If the interaction of E×Pro is significant (p < 0.05), the difference of the treatments (n = 8 per treatment) is tested under –ETECT and +ETEC and labelled accordingly. Otherwise, the values from the Pro treatment (n=16 per treatment) or from ETEC treatment (n = 32) are pooled and then tested under Pro and ETEC, respectively. The treatment difference under Pro is labelled and the difference between –ETEC and +ETEC is significant if the p value of E is < 0.05. These notes are also applied to the following tables. Values within a row with different letters differ significantly (p < 0.05). When the value of ADG became negative, it means that pigs lost weight.

### Blood Parameters

The concentrations of various blood cells (such as red blood cells and white blood cells) often presents the health status of human and animals (Mairbaurl, 2013). The effects of *L. rhamnosus* LB1 on blood cell counts of piglets are summarised in **Table 3**. Briefly, a significant ETEC × probiotic interaction was observed on eosinophilic granulocyte proportion (EOS%) (*p* < 0.05), in addition to a significant trend on monocytes proportion (MONO%) (*p* < 0.10). Oral administration of CT, LL, and HL in piglets significantly reduced the content of Hemoglobin (HGB) in plasma (*p* < 0.05). Among them, oral administration of LL and HL significantly reduced MONO% (*p* < 0.05), while CT had a tendency to reduce MONO% (*p* < 0.10). Compared with the EOS% in the control group, the EOS% in the CT and HL groups significantly decreased (*p* < 0.05), and the EOS% in the LL group tended to decrease (*p* < 0.10).

**Table 3:** Effects of *Lactobacillus rhamnosus* LB1 on blood cell counts of piglets.

Item	- ETEC				+ ETEC				SEM	ETEC		Pro				*P*-value		
	-	CT	LL	HL	-	CT	LL	HL		+	-	-	CT	LL	HL	E	Pro	E×Pro
HGB	125.5	117.9	121.6	127.7	152.5	118.4	130.2	124.0	19.62	131.3	123.2	139.0^b^	118.1^a^	125.9^a^	125.9^a^	0.071	0.014	0.790
MONO %	1.98	2.06	2.03	1.77	2.63	1.96	1.59	1.58	0.63	1.94	1.96	2.31b	2.01ab	1.81a	1.67a	0.882	0.018	0.058
EOS %	5.80^b^	3.86^a^	4.69^ab^	3.60^a^	3.58^a^	4.08^a^	4.02^a^	3.42^a^	1.17	3.76	4.49	4.69	3.93	4.36	3.51	0.004	0.006	0.005
TP g/L	52.24	49.64	53.86	58.53	58.48	54.18	60.09	59.64	5.52	58.10	53.57	55.36b	51.91a	56.97bc	59.08c	<0.001	<0.001	0.317
ALT U/L	44.13^bc^	42.0^bc^	44.75^bc^	46.57^c^	30.63^a^	35.69^ab^	45.71^c^	37.88^abc^	7.92	37.48	44.36	37.38	38.84	45.23	42.22	<0.001	0.003	0.016
ALP U/L	490.4^ab^	492.1^ab^	451.9^ab^	437.6^ab^	375.8^a^	419.1^ab^	575.6^b^	502.9^ab^	110.7	468.4	468.0	433.1	455.6	513.7	470.2	0.988	0.151	0.004
TC mg/dL	68.78	75.98	81.25	87.14	79.24	79.01	86.33	88.78	11.91	83.34	78.29	74.01a	77.49ab	83.79bc	87.96c	0.065	0.002	0.671
GLU mg/dL	83.84^ab^	81.65^ab^	82.20^ab^	85.04^ab^	72.32^a^	75.36^a^	110.90^b^	73.41^a^	24.15	83.00	83.18	78.08	78.51	96.55	79.22	0.974	0.065	0.039
P mg/dL	6.35	6.09	6.86	6.51	6.06	6.30	8.55	6.46	1.49	6.84	6.45	6.20a	6.20a	7.70b	6.48a	0.254	0.007	0.173
CREA mg/dL	75.33^ab^	70.63^a^	71.58^a^	74.66^a^	68.44^a^	73.63^a^	105.8^b^	77.0^ab^	21.51	81.22	73.05	71.88	72.13	88.70	75.83	0.098	0.057	0.024

HGB, haemoglobin; MONO, monocyte; EOS, eosinophil granulocyte; BASO, basophil granulocyte; TP, total protein; ALT, alanine; transaminase; TC, total cholesterol; GLU, glucose; P, phosphorus; CREA, creatinine. Values within a row with different letters differ significantly (p < 0.05).

Similarly, the blood biochemistry can give further information on what have potentially happened behind the physiology of human and animals (Ashraf and Rea, 2017). The effects of *L. rhamnosus* LB1 on plasma biochemical indicators are shown in **Table 3**. Significant ETEC × probiotic interactions were observed on the activity of alkaline phosphatase (ALP) and alanine amino transferase (ALT) as well as the content of glucose (GLU) and creatinine (CREA) (*p* < 0.05). The ETEC infection markedly increased total protein (TP) content and reduced ALT activity compared with none-challenged piglets (*p* < 0.05). The HL supplementations increased the content of total cholesterol (TC) and TP compared with those in none-supplemented piglets (*p* < 0.05). The LL supplementations increased the content of total cholesterol (TC) and phosphorus compared with those in none-supplemented piglets (*p* < 0.05). Among the ETEC infected groups, the LL supplementation significantly increased the activity of ALT and ALP, GLU and CREA content (*p* < 0.05). The HL supplementation had a tendency to increase the activity of ALT and ALP and CREA content (*p* < 0.10). The CT supplementation had a tendency to increase the activity of ALT and ALP (*p* < 0.10).

The results of redox status are summarised in **Table 4**. DAO activity and the content of H_2_O_2_, D-xylose and MDA were found to have significant ETEC × probiotic interactions in this study (*p* < 0.05). The ETEC infection significantly increased H_2_O_2_ content and the activity of DAO compared with those in none-infected piglets (*p* < 0.05). Both the HL and LL supplementation had a tendency to increase the activity of GSH-PX compared with that in none-supplemented piglets (*p* < 0.10). Among the piglets unchallenged with ETEC, the CT and HL supplementations had a tendency to decrease MDA content (*p* < 0.10). The CT supplementation significantly decreased H_2_O_2_ content (*p* < 0.05), while the LL and HL supplementations had a tendency to decrease H_2_O_2_ content (*p* < 0.10). Among the piglets challenged with ETEC, the CT supplementation significantly decreased the content of H_2_O_2_ and D-xylose (*p* < 0.05) and had a tendency to decrease the activity of DAO and MDA content (*p* < 0.10). The supplementation of LL significantly decreased the activity of DAO and MDA content (*p* < 0.05) and had a tendency to decrease the content of H_2_O_2_ (*p* < 0.10), the supplementation of HL significantly decreased the activity of DAO (*p* < 0. 05) and had a tendency to decrease the content of MDA (*p* < 0.10).

**Table 4:** Effects of *Lactobacillus rhamnosus* LB1 on plasma enzymes and related products of piglets.

Item	- ETEC				+ ETEC				SEM	ETEC		Pro				*P*-value		
	-	CT	LL	HL	-	CT	LL	HL		+	-	-	CT	LL	HL	E	Pro	E×Pro
CAT(U/ml)	13.04	7.39	11.05	8.57	11.07	10.74	15.38	10.63	4.20	11.95	10.01	12.06bc	9.06a	13.21c	9.60ab	0.041	0.006	0.095
GSH-Px(μmol/L)	645.8	1209	1007	830.4	705.1	826.9	720.6	942.9	336.9	798.9	923.1	675.5a	1018b	863.6ab	886.7ab	0.109	0.023	0.060
DAO(U/ml)	15.18^a^	16.65^a^	17.43^ab^	23.13^abc^	46.23^d^	32.94^cd^	21.60^abc^	30.96^bc^	12.93	32.93	18.10	30.71	24.79	19.51	27.04	<0.001	0.006	<0.001
MDA(nmol/ml)	12.33^ab^	8.20^a^	12.21^ab^	8.84^a^	14.47^b^	9.89^ab^	8.29^a^	12.76^ab^	3.63	11.35	10.39	13.40	9.04	10.25	10.80	0.211	0.002	0.004
SOD(U/ml)	53.02	53.94	51.24	46.61	49.69	59.85	63.65	49.96	13.86	55.79	51.20	51.36	56.89	57.45	48.28	0.182	0.176	0.437
H_2_O_2_(mmol/ml)	209.9^bc^	88.85^a^	197.1^abc^	110.4^ab^	258.7^c^	103.0^ab^	184.6^abc^	254.6^c^	93.0	200.2	151.6	234.3	95.93	190.9	182.5	0.009	<0.001	0.018
D-xylose(mmol/L)	0.63^bc^	0.61^bc^	0.41^ab^	0.67^c^	0.54^bc^	0.28^a^	0.76^c^	0.61^bc^	0.19	0.55	0.58	0.59	0.45	0.58	0.64	0.427	0.015	<0.001
IL-1β(pg/ml)	121.8^ab^	116.9^a^	124.6^ab^	122.4^ab^	151.8^c^	129.9^ab^	134.9^b^	133.6^b^	13.0	137.6	121.4	136.8	123.4	129.8	128.0	<0.001	0.002	0.017
IL-8(pg/ml)	82.2^b^	70.4^a^	68.2^a^	68.2^a^	59.3^a^	59.2^a^	61.5^a^	64.9^a^	9.72	61.23	72.21	70.73	64.81	64.86	66.48	<0.001	0.114	0.004
TNF-α(pg/ml)	117.1^ab^	103.3^a^	128.4^ab^	112.9^ab^	136.5^b^	99.9^a^	109.4^abc^	122.1^ab^	19.79	117.00	115.40	126.80	101.58	118.91	117.50	0.729	0.002	0.035

CAT, catalase; GSH-Px, glutathione peroxidase; DAO, diamine oxidase; MDA, malondialdehyde; IL, interleukin; TNF, tumour necrosis factor. Values within a row with different letters differ significantly (p < 0.05).

The results of inflammatory parameters are also summarised in **Table 4**. There was a significant interaction of ETEC × probiotic on the content of interleukin-1β (IL-1β), TNF-α and IL-8 (*p* < 0.05). Compared with the control group, the CT group showed a tendency in the reduction of IL-1β and TNF-α (*p* < 0.10) and a significant decrease of IL-8 (*p* < 0.05); IL-8 in the LL and HL groups was also significantly reduced (*p* < 0.05). The ETEC group exhibited a significant increase of IL-1β (*p* < 0.05), and a tendency in the reduction of IL-8 (*p* < 0.1). Compared with the ETEC group, IL-1β and TNF-α in the CT + ETEC group were significantly reduced (*p* < 0.05). While IL-1β in the LL + ETEC group and HL + ETEC group was significantly reduced (*p* < 0.05), TNF-α had a tendency to decrease (*p* < 0.10).

### Intestinal Morphology

**Figure 1:**
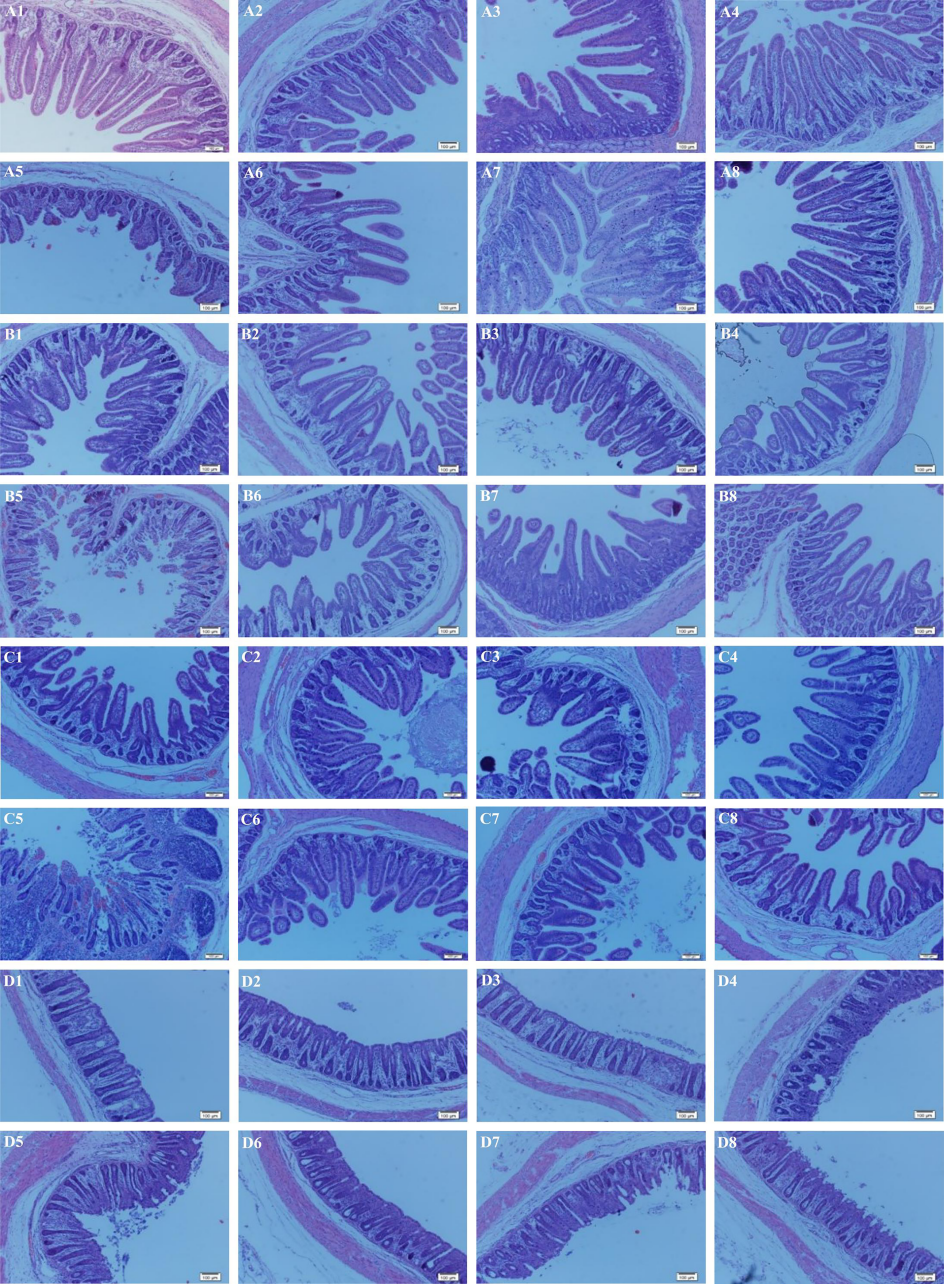
Effects of *Lactobacillus rhamnosus* LB1 on the intestinal morphology of piglets. **(A)** duodenum; **(B)** jejunum; **(C)** ileum; **(D)** colon; (1) control group; (2) CT group; (3) LL group; (4) HL group; (5) ETEC group; (6) CT + ETEC group; (7) LL + ETEC group; (8) HL + ETEC group. Sixty-four tissue slides from each intestinal region per group of the piglets were examined. Representative images are presented.

The microscopic pictures of intestinal mucosal are shown in **Figure 1**. The ETEC infection obviously injured intestinal integrity and villus structure, whereas the supplementation of LB1 remarkably restored the damage caused by ETEC (*p* < 0.05). Results of intestinal mucosal morphology are summarised in **Table 5**. There were significant interactions of ETEC × probiotic on villus height (VH) and VH/crypt depth (CD) in the jejunum and ileum and CD in the colon (*p* < 0.05). Compared with none-challenged piglets, the ETEC infection significantly increased CD in the duodenum, ileum and colon, decreased VH, surface area and ratio of VH/CD in the duodenum, jejunum and ileum (*p* < 0.05). Compared with LB1-none-supplemented piglets, the LL supplementation significantly decreased CD in the duodenum (*p* < 0.05), and the LL and HL supplementations had a tendency to increase villus surface area in the ileum (*p* < 0.10). The CT supplementation significantly increased villus surface area in the ileum (*p* < 0.05) and had a tendency to decrease CD in the duodenum (*p* < 0.10). Compared with the control group, the CT group had significantly increased ileal VH (*p* < 0.05), and the LL and HL groups tended to show increased ileal VH (*p* < 0.10). The ETEC group demonstrated significantly reduced jejunal VH and VH/CD, reduced ileal VH, and significantly increased colon CD (*p* < 0.05). Compared with the ETEC group, the CT + ETEC and LL + ETEC groups had significantly reduced colon CD (*p* < 0.05), which also exhibited a tendency to increase jejunal and ileal VH. The HL + ETEC group showed significantly reduced colon CD, significantly increased ileal VH (*p* < 0.05) and an increased jejunal VH trend (*p* < 0.10).

**Table 5:** Effects of *Lactobacillus rhamnosus* LB1 on the intestinal morphology of piglets.

Item	- ETEC				+ ETEC				SEM	ETEC		Pro				*P*-value			
	-	CT	LL	HL	-	CT	LL	HL		+	-	-	CT	LL	HL	E	Pro	E×Pro
Duodenum																			
VH (µm)	404.4	372.4	354.0	381.1	296.5	341.3	291.8	310.5	67.62	310.0	378.0	350.4	356.9	322.9	345.8	<0.001	0.390	0.332	
VW (µm)	106.1	107.3	101.1	104.8	95.32	100.7	105.9	99.19	12.02	100.3	104.8	100.7	104.0	103.5	102.0	0.139	0.867	0.316	
CD (µm)	117.4	109.0	96.01	117.7	116.9	121.3	113.9	129.4	17.71	120.4	110.1	117.2b	115.2ab	104.9a	123.6b	0.013	0.017	0.434	
VSA	10461	9873	8801	9607	6727	8387	7067	7079	2278	7315	9685	8594	9130	7934	8343	<0.001	0.376	0.366	
VH/CD	3.48	3.33	3.72	3.24	2.48	2.66	2.56	2.57	0.67	2.57	3.44	2.98	2.99	3.14	2.91	<0.001	0.626	0.431	
Jejunum																			
VH (µm)	354.5^c^	274.8^abc^	266.2^abc^	287.3^bc^	228.2^a^	264.8^abc^	284.7^abc^	238.5^ab^	41.55	254.1	283.2	266.4	269.8	275.5	262.9	0.002	0.787	0.002	
VW (µm)	91.49	90.92	94.56	93.56	87.18	86.08	84.85	77.74	9.10	83.96	92.63	89.33	88.50	89.70	85.65	<0.001	0.467	0.153	
CD (µm)	94.60	106.9	105.5	103.6	102.5	107.1	109.7	110.5	12.01	107.5	102.7	98.55	107.0	107.6	107.1	0.108	0.097	0.789	
VSA	6849	6648	6644	6532	4649	5641	4868	4740	1319	4975	6668	5749	6144	5756	5636	<0.001	0.522	0.421	
VH/CD	3.34^c^	2.75^abc^	2.53^ab^	2.95^bc^	2.24^ab^	2.55^ab^	2.60^abc^	2.18^a^	0.57	2.39	2.89	2.79	2.65	2.56	2.57	<0.001	0.514	0.004	
Ileum																			
VH (µm)	301.6^bc^	352.4^d^	315.3^cd^	314.5^cd^	260.1^a^	293.8^abc^	268.4^ab^	316.2^cd^	36.56	284.6	321.0	280.9	323.1	291.9	315.4	<0.001	<0.001	0.008	
VW (µm)	89.01	99.30	93.37	88.70	86.57	93.93	88.01	95.66	12.17	91.04	92.59	87.79	96.61	90.69	92.18	0.611	0.231	0.433	
CD (µm)	92.0	99.48	88.19	92.40	94.95	99.35	93.70	110.0	10.99	99.50	93.02	93.47ab	99.41bc	90.94ab	101.2c	0.009	0.010	0.057	
VSA	6609	8151	7071	6687	5205	6307	5453	6742	1508	5927	7130	5907a	7229b	6262ab	6714ab	0.001	0.035	0.173	
VH/CD	3.25	3.55	3.61	3.48	2.68	2.97	2.91	2.76	0.51	2.83	3.47	2.96	3.26	3.26	3.12	<0.001	0.132	0.927	
Colon																			
CD (µm)	117.5^a^	126.7^ab^	124.7^ab^	128.9^ab^	264.1^e^	175.7^d^	145.2^bc^	158.9^cd^	47.63	186.0	124.5	190.8	151.2	135.0	143.9	<0.001	<0.001	<0.001	

VH, villus height; CD, crypt depth; VSA, villus surface area. Values within a row with different letters differ significantly (p < 0.05).

### Regulation of Gene Expression

The profile of relative expression levels of genes is summarised in **Table 6**. Significant interactions of ETEC × Probiotic were observed on expressions of genes IL-8, HSP70, S100 calcium binding protein A12 (S100A12), myxovirus resistance 1 (Mx1), myxovirus resistance 2 (Mx2), nuclear factor erythrocyte two related factors-2 (Nrf2), glutathione peroxidas 2 (GPX2) and Arginase1 (ARG1) in the jejunum and ileum, as well as haptoglobin (HP) expression in the jejunum (*p* < 0.05). Compared with none-challenged piglets, the ETEC infection significantly increased expression levels of IL-8, HP and HSP70 (*p* < 0.05), decreased expression levels of Mx1, Mx2 and GPX2 in the jejunum (*p* < 0.05). In the ileum, the ETEC infection significantly increased expression levels of HP, HSP70, S100A12, Nrf2 and ARG1, and decreased expression levels of Mx2 and GPX2 (*p* < 0.05).

**Table 6:** Effects of *Lactobacillus rhamnosus* LB1 on gene expression in the jejunum and ileum of piglets.

Item	- ETEC				+ ETEC				SEM	ETEC		Pro				*P*-value		
	-	CT	LL	HL	-	CT	LL	HL		+	-	-	CT	LL	HL	E	Pro	E×Pro
Jejunum																		
IL-8	1.000^abc^	0.757^a^	0.986^abc^	1.260^cd^	1.448^d^	1.101^abcd^	1.141^bcd^	0.891^ab^	0.080	1.145	1.001	1.224	0.929	0.778	1.075	0.014	0.006	<0.001
HP	1.000^a^	0.814^a^	1.079^a^	1.694^b^	1.809^b^	0.953^a^	1.697^b^	0.782^a^	0.097	1.310	1.147	1.405	0.883	0.964	1.238	0.021	<0.001	<0.001
HSP70	1.000^a^	1.674^bc^	1.243^ab^	1.323^abc^	1.072^a^	1.168^a^	1.775^cd^	2.179^d^	0.111	1.548	1.310	1.036	1.421	1.065	1.751	0.004	<0.001	<0.001
S100A12	1.000^e^	0.446^abc^	0.542^bcd^	0.723^d^	1.528^f^	0.352^ab^	0.697^cd^	0.259^a^	0.059	0.709	0.678	1.264	0.399	0.445	0.491	0.463	<0.001	<0.001
Mx1	1.000^bcd^	1.023^bcd^	1.113^cd^	1.174^d^	0.446^a^	0.845^bc^	0.298^a^	0.772^b^	0.070	0.590	1.078	0.723	0.934	0.631	0.973	<0.001	<0.001	<0.001
Mx2	1.000^bc^	0.985^bc^	1.272^c^	0.809^b^	0.350^a^	1.249^c^	0.684^b^	1.288^c^	0.073	0.893	1.017	0.675	1.117	0.807	1.049	0.019	<0.001	<0.001
Nrf2	1.000^c^	0.467^a^	0.578^ab^	0.637^ab^	0.994^c^	0.604^ab^	0.705^b^	0.496^ab^	0.053	0.700	0.670	0.997	0.536	0.465	0.566	0.440	<0.001	0.041
GPX2	1.000^c^	0.735^b^	1.310^d^	1.546^d^	0.584^ab^	0.431^a^	0.693^b^	0.377^a^	0.058	0.521	1.148	0.792	0.583	0.828	0.962	<0.001	<0.001	<0.001
ARG1	1.000^c^	1.187^c^	0.653^ab^	0.444^a^	1.698^d^	0.876^bc^	0.384^a^	0.486^a^	0.073	0.861	0.821	1.349	1.031	0.423	0.465	0.446	<0.001	<0.001
Ileum																		
IL-8	1.000^bcd^	0.634^a^	1.041^bcd^	1.224^d^	0.930^abcd^	0.805^ab^	1.115^cd^	0.897^abc^	0.069	0.937	0.975	0.965	0.719	0.799	1.061	0.433	<0.001	0.004
HP	1.000	0.743	0.798	0.883	1.226	1.170	0.972	1.002	0.071	1.093	0.856	1.113^b^	0.957^a^	0.885^a^	0.942^a^	<0.001	0.014	0.158
HSP70	1.000^bc^	0.778^ab^	0.774^ab^	0.587^a^	1.041^c^	0.786^ab^	0.879^bc^	0.930^bc^	0.057	0.909	0.785	1.021	0.782	0.607	0.759	0.003	<0.001	0.024
S100A12	1.000^c^	0.259^a^	0.531^ab^	1.055^c^	2.352^d^	0.535^ab^	0.806^bc^	0.397^a^	0.088	1.023	0.712	1.676	0.397	0.467	0.726	<0.001	<0.001	<0.001
Mx1	1.000^c^	0.550^a^	0.680^ab^	0.896^bc^	0.707^ab^	0.883^bc^	0.554^a^	1.051^c^	0.060	0.799	0.782	0.854	0.716	0.478	0.973	0.689	<0.001	<0.001
Mx2	1.000^d^	0.581^bc^	0.634^c^	0.453^b^	0.057^a^	0.090^a^	0.070^a^	0.136^a^	0.037	0.090	0.667	0.529	0.335	0.336	0.295	<0.001	<0.001	<0.001
Nrf2	1.000^bc^	0.645^a^	0.742^ab^	0.826^ab^	1.635^d^	1.184^c^	1.003^bc^	0.974^abc^	0.077	1.199	0.803	1.318	0.915	0.622	0.900	<0.001	<0.001	0.008
GPX2	1.000^b^	0.509^a^	1.094^b^	0.964^b^	0.692^a^	0.592^a^	0.620^a^	0.612^a^	0.058	0.629	0.892	0.846	0.551	0.702	0.788	<0.001	<0.001	<0.001
ARG1	1.000^b^	0.630^a^	0.743^ab^	0.836^ab^	1.515^c^	1.409^c^	1.593^c^	0.767^ab^	0.081	1.321	0.802	1.257	1.020	0.770	0.801	<0.001	<0.001	<0.001

HP, haptoglobin; HSP70:Heat Shock Protein 70; S100A12:S100 calcium binding protein A12; Mx1, myxovirus resistance 1; Mx2, myxovirus resistance 2; Nrf2, nuclear factor erythrocyte two related factors-2; GPX2, glutathione peroxidas 2; ARG1, Arginase1. Values within a row with different letters differ significantly (p < 0.05).

Compared with the control group, the CT group had significantly reduced expression of Nrf2 and S100A12 (*p* < 0.05) and a tendency to reduce the expression of IL-8 in the jejunum (*p* < 0.10), and significantly reduced expression levels of IL-8, S100A12, Nrf2 and ARG1 in the ileum (*p* < 0.05). There was also a tendency in reducing the expression of HSP70 in the ileum (*p* < 0.10). In the LL group compared with the control group of piglets, there were significantly reduced expression of Nrf2, S100A12 and ARG1 and an increased expression of GPX2 in the ileum (*p* < 0.05) with a tendency in increasing the expression of Mx1, Mx2 (*p* < 0.10) in the jejunum. A significant reduction in the expression of S100A12 (*p* < 0.05) and a tendency to reduce the expression of HSP70, Nrf2 and ARG1 (*p* < 0.10) were also detected in the ileum. In the HL group, significantly reduced expression levels of S100A12, Nrf2 and ARG1 and increased expression of GPX2 were observed in the jejunum. In the ileum, the expression of HSP70 was significantly reduced (*p* < 0.05), and the expression levels of Nrf2 and ARG1 tended to decrease (*p* < 0.10). The ETEC group demonstrated significantly increased expression levels of IL-8, HP, S100A12 and ARG1, and reduced expression of Mx1, Mx2 and GPX2 in the jejunum (*p* < 0.05). In the ileum, the expression levels of S100A12, Nrf2 and ARG1 were significantly increased, and the expression levels of Mx1, Mx2 and GPX2 were significantly reduced (*p* < 0.05).

Compared with the ETEC group, the CT + ETEC group had significantly reduced expression levels of HP, S100A12, ARG1, and Nrf2, and increased expression of Mx1 and Mx2 in the jejunum (*p* < 0.05). In the ileum, the CT + ETEC group exhibited significantly reduced expression of HSP70, S100A12, and Nrf2 (*p* < 0.05), and a tendency to reduce the expression of IL-8 (*p* < 0.10). In the LL + ETEC group, there were significantly reduced expression levels of S100A12, ARG1 and Nrf2 and increased expression of Mx2 (*p* < 0.05), and a decreased trend in the expression of IL-8 in the jejunum (*p* < 0.10). In the ileum, the expression of S100A12 and Nrf2 was significantly reduced (*p* < 0.05), while the expression HSP70 showed a tendency to decrease (*p* < 0.10). In the HL + ETEC group, while the expression levels of IL-8, HP, S100A12, Nrf2 and ARG1 were significantly reduced in the jejunum (*p* < 0.05), the expression levels of Mx1 and Mx2 were significantly increased (*p* < 0.05). In the ileum, the expression levels of S100A12, Nrf2 and ARG1 were significantly reduced and the expression of Mx1 was significantly increased (*p* < 0.05). The expression of HP and HSP70 exhibited a tendency to decrease (*p* < 0.10).

### Regulation of Protein Production

**Figure 2:**
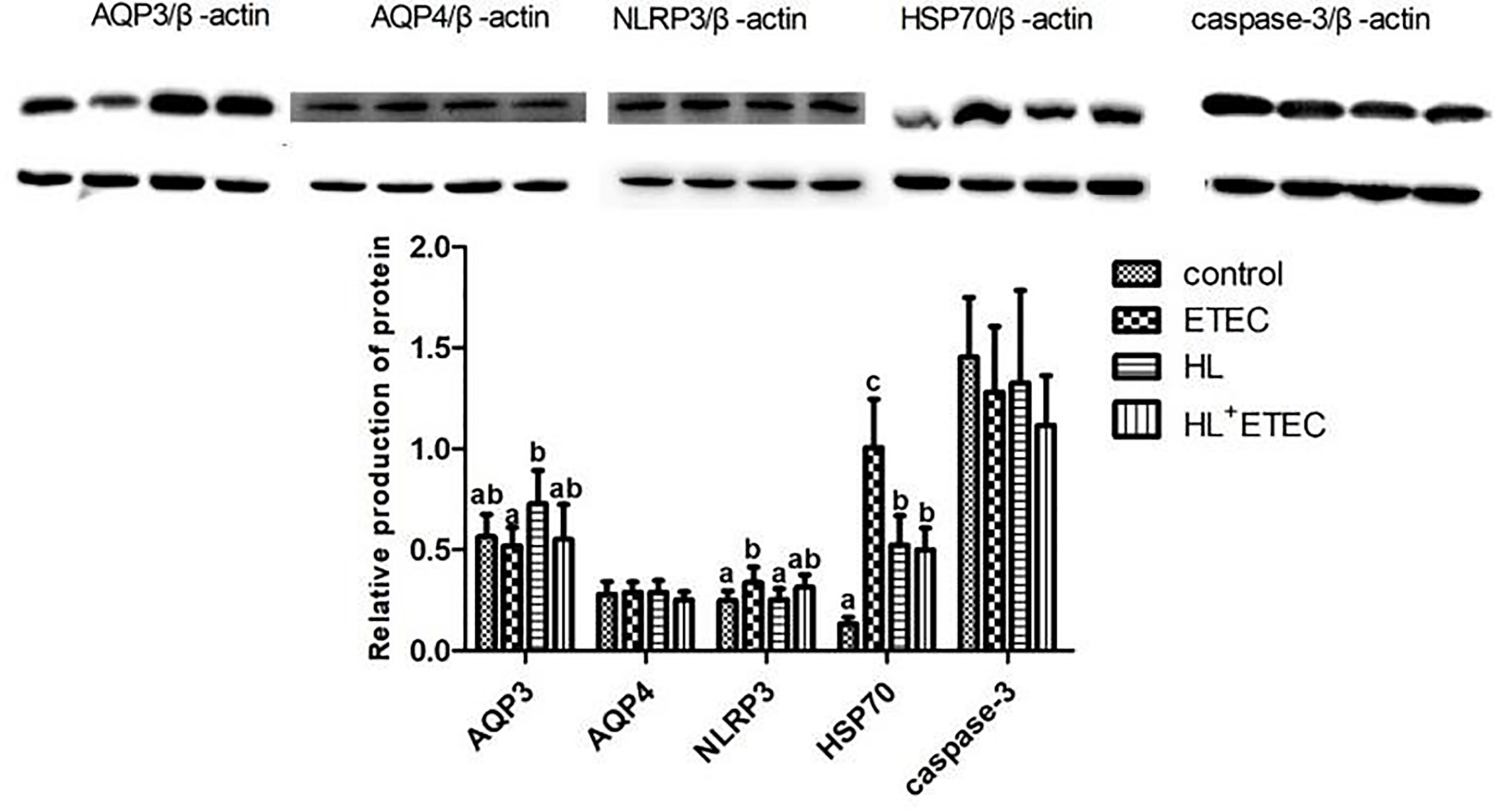
Effects of *Lactobacillus rhamnosus* LB1 on protein production in the jejunum of piglets Values among different treatments with different letters differ significantly (p < 0.05).

The profiles of relative levels of protein production in the jejunum and ileum are summarised in **Figure 2**. Compared with the control group (basal diet), the ETEC infection significantly increased the production of NLRP3 and HSP70 (*p* < 0.05), and the HL treatment significantly increased the production of HSP70 (*p* < 0.05), which was much lower than that in the ETEC group (*p* < 0.05). The production of AQP3 in the HL group showed a tendency to increase (*p* < 0.10). Compared with the ETEC group, the HL+ETEC group exhibited significantly reduced production of NLRP3 and HSP70 and a tendency to increase the production of AQP3.

## Discussion

Piglet diarrhoea is one of the major problems in swine production, which causes huge economic losses to the swine industry. The primary pathogenic bacterium causing piglet diarrhoea is ETEC, whose virulence depends on the co-effect of enterotoxins and adhesion. A marked finding from the present study is the significant reduction of ETEC-induced diarrhoea and mortality rate by LB1 supplementation, indicating that *L. rhamnosus* could effectively relieve diarrhoea in lactating piglets.

Intestinal health is critical for the healthy growth of animals and well-being of humans (Wu, 2010). Intestinal integrity is an important manifestation of intestinal health, such as surface area, villus height, the ratio of villus height to crypt depth and crypt depth as the intestinal morphology indexes can reflect intestinal morphologic development and intestinal morphological integrity. Generally, the increases of the intestinal morphology indexes such as villus surface area, the ratio villus/crypt as well as villus height reflect the improvement of intestinal health and intestinal absorption capacity (Pluske et al., 1996). In the present study, ETEC infection undermined the integrity of the intestinal tract, leading to severe intestinal damage of piglets, while the supplementation of *L. rhamnosus* LB1 alleviated the intestinal injury caused by ETEC, demonstrating beneficial effects on intestinal integrity.

DAO activity as a non-invasive biomarker is frequently used to reflect changes in the structure and function of the intestinal mucosa (Agúndez et al., 2012). When the cells in the intestinal mucosa experience necrosis and slough off into the enteric enterocele under certain conditions, DAO levels in the intestinal mucosa is declined and DAO levels in circulation is increased (Kitanaka et al., 2002). In the present study, LB1 supplementation decreased blood DAO, indicating that *L. rhamnosus* could restore the damage of intestinal barrier caused by ETEC.

AQP and Gut ion transporters may be potential targets for the prevention and treatment of diarrhoea in animals and humans (Ikarashi et al., 2016). The gene knockout mice of AQP3 have a mucosal innate immune disorder (Thiagarajah et al., 2017). NLRP3 is a subfamily of NLRs, called inflammatory bodies, which can respond to a variety of microorganisms, environmental stimuli and host cell damage, and regulate intestinal homeostasis and inflammatory responses through these pathways (Sellin et al., 2015; Wu et al., 2016). HSP70 is the first protein that responds to heat stress in the liver and is accompanied by haemorrhagic shock (Abdelnour et al., 2019). By increasing the expression level of HSP70, the animal body promotes protein folding and prevents the deformation of protein aggregation. In the present study, *L. rhamnosus* supplementation decreased the gene expression of NLRP3 and HSP70, increased AQP expression, which may be one of the primary mechanisms to alleviate intestinal mucosal injury and diarrhoea caused by ETEC.

The capacity of the biosystem to readily detoxify the reactive intermediaries and the unbalance between the systematic phenomenon of reactive oxygen species can be reflected by oxidative stress (Cabello-Verrugio et al., 2016), whereas cells can protect themselves from oxygenates and other hydroxyl radicals by antioxidant enzymes (including GSH-Px, CAT and SOD) (Harris, 1992; Yu, 1994). MDA as a marker is usually used to evaluate the level of oxidative stress (Del Rio et al., 2005). The main product of oxidative stress is H_2_O_2_ in the body (Sies, 2014). Results in this study implied that the supplementation of *L. rhamnosus* could alleviate oxidative stress induced by ETEC and improve anti-oxidative capacity.

IL-1β, IL-8, and TNF-α have a synergistic and inducing relationship, which are responsible for regulating and mediating the body’s immune function. With the activation of macrophages, the body first secretes a large amount of TNF-α, then promotes the production of IL-1β, increases cell permeability, promotes the synthesis of adhesion molecules, and releases inflammatory mediators to white blood cells. The primary function of IL-8 is to induce neutrophil chemotaxis, and also induce another granulocyte chemotaxis, causing them to migrate to the infection site, thereby playing a phagocytic effect (Moser and Loetscher, 2001; Stillie et al., 2009). The expression of IL-8 promotes the aggregation of neutrophils and activates neutrophils to release various active substances. Although this physiological activity can cause cell damage, it can also play a bactericidal role (Wang H. Y. et al., 2011). Some related *in vitro* studies have shown that lactic acid bacteria can alleviate the inflammation caused by pathogenic bacteria, *e.g. L. plantarum*, and *L. rhamnosus* can inhibit the expression of pro-inflammatory cytokines TNF-α and IL-1β induced by *E. coli* (Li et al., 2012; Chytilová et al., 2013). In this study, ETEC infection significantly increased the content of IL-1β and IL-8, whereas *L. rhamnosus* LB1 supplementation decreased the levels of TNF-α and IL-1β, suggesting its effects on alleviation of the inflammation and enhancement of host immunity.

Many cytokines are essential for intestinal function (Roselli et al., 2007). IL-8 can bind to CXCR1 and CXCR2 receptors with high affinity, and as the primary chemokine of neutrophils, it plays a vital role in the initiation and maintenance of inflammatory response (Crespo et al., 2018). S100A12 found in mammalian tissues is considered to be an indicator for the diagnosis of inflammatory responses (Zhong et al., 2018). S100A12 is mainly expressed in monocytes and neutrophils, which is associated with innate immune responses. Some studies have shown that S100A12 is increased in certain human inflammatory diseases (Däbritz et al., 2013; Hofmann-Bowman et al., 2017). The levels of genes encoding antiviral proteins, especially 2’-5 ‘oligoadenylate synthetase (OAS) and myxovirus (MX) can be induced by IFN-α/β (García-Sastre and Biron, 2006; Takaoka and Yanai, 2006). Nuclear factor-erythrocyte-associated factor 2 (Nrf2) is a transcriptional regulatory element that maintains the cell’s redox state by promoting antioxidant response participants (glutathione peroxidase et al.) to the cell protection and antioxidant gene promoters. Under normal circumstances, the concentration of Nrf2 in the cytoplasm is low. When the animal body is subjected to oxidative stress, Nrf2 will transfer to the nucleus and activate related enzymes in the nucleus, such as the combination of antioxidant response elements, thereby activating the transcription of many protective genes. These protective genes play a role in maintaining the redox balance and exert anti-inflammatory and anti-oxidant effects (Niture et al., 2014). In addition, a recent study has shown that the concentration of Nrf2 is steadily increased in the cancerous tissues and organs detected (Kahroba et al., 2019). Among the antioxidants, GPX, CAT, and SOD are the first protections in antioxidant stress (Zhu et al., 2012). GPX is responsible for catalyzing the reduction of hydrogen peroxide and protecting tissues from damage by ROS. Previous studies have shown that other plant extracts (quercetin and rutinand) and polyphenols (hypericin, rotenone) can significantly induce the expression of SOD, CAT, and GPX genes (Sánchez-Reus et al., 2007; Manubolu et al., 2014; El-Sayed et al., 2017). In this study, the supplementation of *L. rhamnosus* LB1 had opposite effects on regulating expressions of genes related to inflammation and anti-oxidation, which may be one of the primary mechanisms to alleviate the inflammatory response and oxidative stress caused by ETEC and improve intestinal health.

## Conclusion

Supplementation of *L. rhamnosus* LB1 relieved diarrhoea, intestinal mucosal damage, inflammatory and oxidative stress, and improved intestinal integrity, immune and anti-oxidative capacity. The beneficial effects appeared to be regulated by changing the gene expression and protein production of the functionally related molecules.

## Data Availability Statement

The original contributions presented in the study are included in the article/**Supplementary Material**. Further inquiries can be directed to the corresponding authors.

## Ethics Statement

The animal study was reviewed and approved by the Animal Care and Use Committee of Wuhan Polytechnic University (Wuhan, Hubei, China) (2018-0625, 25th Jun 2018).

## Author Contributions

TW, YH, and JG conceived and designed the experiments. YS, MZ, LZ, and ZM performed the experiments. TW, YS, DZ, LW, HY, and YZ analysed the data. TW, YH, YS, JG, and YZ wrote the paper. All authors contributed to the article and approved the submitted version.

## Funding

This research was supported by the Open Project of Hubei Key Laboratory of Animal Nutrition and Feed Science (Grant number 201905), National Key R&D Program of China (2017YFD0500505), Hubei Provincial Technology and Innovation Program (2017AHB062), Agriculture & Agri-Food Canada through the A-base program (AAFC Project ID: J-001391), Swine Science Cluster Program III (AAFC Project ID: J-002109), the Open Project of Hubei Key Laboratory of Animal Nutrition and Feed Science (grant number DKXY2020002) and Basic research of Chinese Academy of Fishery Sciences operating expenses(2018JB F3).

## Conflict of Interest

The authors declare that the research was conducted in the absence of any commercial or financial relationships that could be construed as a potential conflict of interest.

## Publisher’s Note

All claims expressed in this article are solely those of the authors and do not necessarily represent those of their affiliated organizations, or those of the publisher, the editors and the reviewers. Any product that may be evaluated in this article, or claim that may be made by its manufacturer, is not guaranteed or endorsed by the publisher.

## Supplementary Material

Supplementary Table 1 Physiological and fermentation properties of *Lactobacillus rhamnosus* LB1. +, Detected; -, Not detected.
