# Supplementary material for: Lactobacillus rhamnosus LB1 Alleviates Enterotoxigenic Escherichia coli-Induced Adverse Effects in Piglets by Improving Host Immune Response and Anti-Oxidation Stress and Restoring Intestinal Integrity
Source: Front Cell Infect Microbiol. 2021 Nov 2;11:724401. doi: 10.3389/fcimb.2021.724401 (PMC8594739; doi:10.3389/fcimb.2021.724401)
Supplement: Supplementary Table 1 — Physiological and fermentation properties of Lactobacillus rhamnosus LB1. +, Detected; -, Not detected. [file Table_1.docx]

Table S1. Physiological and fermentation properties of *Lactobacillus rhamnosus* LB1

| Tested item | Result | Tested item | Result |
| --- | --- | --- | --- |
| Catalase | - | Salicin fermentation | + |
| Oxidase | - | Sucrose fermentation | + |
| Anaerobic growth | + | Xylose fermentation | - |
| Growth at 15 °C | + | Mannitol fermentation | + |
| Hydrolysis of esculin | + | Mannose fermentation | + |
| Nitrate reduction | - | Melezitose fermentation | - |
| Arabinose fermentation | - | Melibiose fermentation | - |
| Cellobiose fermentation | + | Raffinose fermentation | + |
| Fructose fermentation | + | Rhamnose fermentation | + |
| Galactose fermentation | + | Ribose fermentation | + |
| Glucose fermentation | + | Sorbose fermentation | + |
| Lactose fermentation | + | Trehalose fermentation | + |
| Maltose fermentation | + |  |  |

+: Detected; -: Not detected.
